# Polycystic ovary syndrome is linked with the fat mass obesity (FTO) gene variants rs17817449 and rs1421085 in western Saudi Arabia

**DOI:** 10.6026/97320630017904

**Published:** 2021-11-30

**Authors:** Sherin Bakhashab, Asma A Batarfi, Najlaa Filimban, Osama S Bajouh, Ashraf Dallol, Mohammed H Alqahtani

**Affiliations:** 1 Biochemistry Department, King Abdulaziz University, P.O. Box 80218, Jeddah 21589, Saudi Arabia; 2 Centre of Innovation in Personalized Medicine, King Abdulaziz University, P.O. Box 80216, Jeddah 21589, Saudi Arabia; 3 King Faisal Specialist Hospital and Research Center, Clinical Genomics, Department of Genetics, P.O. Box 3354, Riyadh 11211, Saudi Arabia; 4 Department of Obstetrics and Gynaecology, Faculty of Medicine, King Abdulaziz University, P.O. Box 80205, Jeddah 21589, Saudi Arabia; 5 Department of Medical Laboratory Technology, Faculty of Applied Medical Sciences, King Abdulaziz University, P.O. Box 80216, Jeddah 21589, Saudi Arabia; 6 Center of Excellence in Genomic Medicine Research, King Abdulaziz University, P.O. Box 80216, Jeddah 21589, Saudi Arabia

**Keywords:** BMI, Polycystic ovary syndrome, FTO, Single nucleotide polymorphism

## Abstract

Polycystic ovary syndrome (PCOS) is characterised by infertility, obesity, insulin resistance and clinical and/or biochemical signs of hyperandrogenism. Obesity is known to be correlated with PCOS causing ovulatory dysfunction and hormone imbalances. Moreover, fat mass and the obesity gene (FTO) were linked with obesity and PCOS. Therefore, it is of interest to determine the genotype and allele frequency for three FTO variants - rs17817449 (G/T), rs1421085 (C/T) and rs8050136 (A/C) -in western Saudi population. 95 PCOS patients and 94 controls were recruited for this study. The genetic variants were assayed using real-time polymerase chain reaction using TaqMan genotyping assays. The chi-squared test was applied to investigate the difference between single nucleotide polymorphisms on PCOS and control subjects, and binary logistic regression was used to determine the association of FTO variants with PCOS symptoms. Variants rs17817449 and rs1421085 were significantly linked with PCOS susceptibility in the study population. Rs17817449 and rs8050136 were significantly associated with hair loss in the PCOS group. Furthermore, rs1421085 and rs8050136 were associated with a high body mass index (BMI>30 kg/m2). Risk alleles in our population associated with hair loss and elevated BMI in women with PCOS were homozygous C for rs8050136. This data will help in defining the genetic predisposition of PCOS among women in western Saudi Arabia.

## Background:

Polycystic ovary syndrome (PCOS) is a heterogeneous endocrine disorder affecting 4-21% of reproductive age women depending on the diagnostic criteria used [1,2]. Both genetic and environmental factors are associated with the aetiology of this syndrome [3]. The most common metric used in the diagnosis of PCOS is the Rotterdam criteria [4-6], which define PCOS as the presence of two out of the following three symptoms: oligo/anovulation (OA), hyperandrogenism (HA) and polycystic ovarian morphology (PCOM) [7]. Women faces serious complications related to infertility, menstrual cycle irregularity, obesity, stress and variation in hormone levels [8]. These include a high luteinising hormone (LH) to follicle-stimulating hormone (FSH) ratio [7] and serum anti-Mullerian hormone (AMH) [9].

Two genome-wide association studies (GWAS) for PCOS were conducted in Han Chinese populations in which researchers identified 15 risk SNPs from 11 loci: thyroid adenoma associated (THADA), luteinising hormone/choriogonadotropin receptor (LHCGR), follicle-stimulating hormone receptor (FSHR), chromosome 9 open reading frame 3 (C9orf3), differentially expressed in normal and neoplastic cells (DENN) domain-containing 1A (DENND1A), Yes-associated protein 1 (YAP1), Ras-related protein 5B (RAB5B), insulin receptor (INSR), TOX transcriptional coactivator of the p300/CBP-mediated transcription complex (TOX3), SUMO1 pseudogene 1/zinc finger protein 217 (SUMO1P1/ZNF217) and high mobility group AT-hook 2 (HMGA2) [10, 11]. Another large-scale GWAS of a population with European Caucasian ancestry used dense imputation of genotyping to identify six genetic loci associated with PCOS: ErbB2 receptor tyrosine kinase 4/Hairy-related 4 (ERBB4/HER4), YAP1, THADA, follicle-stimulating hormone beta-subunit (FSHβ), double-strand break repair protein (RAD50) and ketopantoate reductase 1 (KRR1) [12]. In addition, four studies of European ancestry populations have confirmed the association of some of these loci with PCOS, including FSHR/LHCGR, DENND1A, RAB5B and THADA [13-16]. GWAS have also revealed the genetic predisposition and susceptibility loci for several genetic diseases, including obesity and diabetes [17-20]. These studies have detected variations in or near the fat mass and obesity gene (FTO), GNPDA2, INSIG2, KCTD15, MC4R, MTCH2, NEGR1, SH2B1 and TMEM18 as susceptibility loci for obesity [17,18,21]. FTO and MC4R were reported to be associated with obesity in PCOS patients [17,18,21-23]. Previously, we had detected the association between MC4R variants rs12970134 and rs17782313 and obese PCOS patients in western Saudi Arabia [24].

FTO is a large gene, approximately 410 kb, located on chromosome 16q12.2. Many GWAS have shown the correlation between variants within the FTO gene and obesity and traits related to obesity [25]. Moreover, the association between FTO and cerebral insulin sensitivity illustrated that FTO affects adiposity and the satiety signal of insulin in the brain and deregulates food intake and thus, body mass index (BMI) [26, 27]. The mechanistic basis for the genetic association between FTO and obesity appears to be the disruption of AT-rich interaction domain 5B repressor through the causal variants of FTO. This disruption results in loss of binding and activation of downstream targets Iroquois homeobox gene 3 (IRX3) and IRX5 during early adipocyte differentiation [28]. FTO single nucleotide polymorphisms (SNPs) rs17817449, rs1421085 and rs8050136, were determined to be associated with obesity in women with PCOS [23]. Other studies showed the direct association between FTO rs8050136 with PCOS development among Chinese women above a specific BMI [29,30]. In addition, Song et al. revealed an association between PCOS susceptibility and hyperandrogenemia with FTO variants rs17817449, rs1421085 and rs8050136 in the Korean population [31]. This study showed that woman (total subjects, PCOS and control) with the G/G genotype in rs17817449, C/C genotype in rs1421085, and A/A genotype in rs8050136 experiencing higher BMI and higher hyper androgenism than women with other genotypes. The G allele in FTO variant rs17817449 was considered a risk allele for obesity and obesity-related traits [32]. The C allele in rs1421085 has also been proposed as a risk factor for obesity and type 2 diabetes mellitus (T2DM) [33,34]. In addition, several studies have reported that the A allele in FTO rs8050136 is a risk factor associated with high BMI and T2DM [22,35,36]. There is no known data on the impact of FTO polymorphisms on PCOS incidence among the Saudi population. Therefore, it is of interest to determine the correlation between FTO gene variants rs8050136, rs17817449 and rs1421085 as risk factors for PCOS and obesity and their contribution to the severity of the disorder using association studies with different clinical PCOS variables. This will help in identifying genetic predispositions for PCOS and specific risk variants associated with this syndrome.

## Materials & Methods:

### Study design:

This case-control observational study included 98 PCOS patients diagnosed according to the Rotterdam Criteria and 98 women with normal ovulation. Poor quality samples were excluded, providing a final sample total of 95 cases and 94 controls. The clinical samples were collected in the Obstetrics and Gynaecology Clinics, King Abdulaziz University Hospital and the Centre of Innovation in Personalized Medicine (CIPM), KAU, Jeddah, Kingdom of Saudi Arabia. The exclusion criteria, power calculation and serum hormonal assays were previously described.[24] Transvaginal or abdominal ultrasound was performed on days 2-4 of the menstrual cycle using a SonixTouch machine (Ultrasonix Medical Corporation, Richmond, BC, Canada). HA was defined as the presence of hirsutism, acne or androgenic alopecia [37,38]. The Biomedical Ethics Unit, Faculty of Medicine, KAU (approval number: 407-15) approved this study and written informed consent was obtained from participants prior to sample collection. The study was conducted in accordance with the Declaration of Helsinki.

### Genotyping:

The QIAamp DNA Mini Blood Kit (Qiagen, Hilden, Germany) was used to isolate DNA from peripheral blood according to the manufacturer's instructions. TaqMan® Open Array® Genotyping Real-time PCR Assays (ThermoFisher Scientific, Waltham, MA, USA) were used to identify the FTO variants rs17817449 (assay ID: C_34511515_10) and rs1421085 (assay ID: C_8917103_10) using 50 ng/µL of DNA. The FTO variant rs8050136 (assay ID: C_2031259_10) was genotyped by the TaqManTM SNP Genotyping Assay (ThermoFisher Scientific) with a DNA concentration of 1-20 ng/µL. Allelic PCR products were analysed using the QuantStudio 12K Flex Real-Time PCR System (ThermoFisher Scientific).

### Statistical analysis:

Quantitative data are presented as median ± inter quartile range (IQR), and p-values were calculated using the Mann-Whitney test as the data followed a non-normal distribution. Qualitative data were described as frequencies. Genotype and allele frequencies were calculated within our cohort population by Hardy-Weinberg equilibrium, and a chi-squared test was used to detect the association between SNPs within the study groups. In addition, the variation in qualitative variables and SNPs was analysed by the chi-squared test. Differences between SNPs and continuous variables were tested using the Mann-Whitney or Kruskal-Wallis tests. The association of FTO variants with PCOS symptoms was then analyzed using binary logistic regression to determine the risk of alleles and odds ratio (OR). For all results, p<0.05 was considered statistically significant. Data analysis was performed using the IBM SPSS software version 24 (SPSSTM Inc., Armonk, NY, USA).

## Results:

The subjects' clinical characteristics are provided in our previous study [24].

## Allele and genotype frequency:

The distribution of the FTO variants' rs17817449, rs1421085 and rs8050136 genotype and allele frequencies is reported in Table 1(see PDF). There was a direct and significant association between PCOS and FTO SNPs rs17817449 and rs1421085 (p<0.0001 and p=0.003, respectively), but no association was detected with FTO rs8050136. Also, there was no association between PCOS and the allele frequency of all SNPs.

## The association of FTO variants rs17817449, rs1421085 and rs8050136 with PCOS clinical characteristics:

There was a significant correlation between FTO variants rs17817449 and rs8050136 and loss of hair in the PCOS group (p=0.038 and p=0.013, respectively) as calculated by the chi-squared test (Table 2 - see PDF). There was a significant difference in BMI between FTO variant rs1421085 genotypes in the PCOS group (p=0.041, Table 2 - see PDF), and the difference was detected between C/C and T/T using Kruskal-Wallis pairwise comparison. However, there were no significant associations nor differences between the FTO variants and other clinical variables such as HA, hirsutism, acne, PCOM, OA, AMH, LH, and FSH. Furthermore, patients with the C/C genotype were more likely to suffer from hair loss (OR=11.88, p<0.0001) and elevated BMI (OR=1.246, p=0.005) than the control with C/C genotype in the rs8050136 variant using binary logistic regression test. Therefore, homozygous C is a risk genotype associated with hair loss and high BMI (Table 3 - see PDF). No significant correlation with other variants and study variables was detected in the PCOS group. Furthermore, adjusted binary logistic regression analysis determined no significant effect of the combined genotypes of different studied variants on PCOS susceptibility.

## Discussion:

In this genetic association study, we examined the FTO variants rs8050136, rs17817449 and rs1421085 for their contribution as risk factors in PCOS. Women with PCOS have elevated rates of obesity compared with non-PCOS women, which influences the severity and prevalence of PCOS [39]. The FTO gene was reported in a GWAS to be associated with obesity and obesity traits [25]; therefore, these variants were selected due to their importance as adiposity mediators. To the best of our knowledge, this is the first study reporting the effect of FTO variants on the risk of PCOS in the Kingdom of Saudi Arabia. In our study population, the frequency of the common T allele of rs17817449 was 39.47% for the PCOS group and 45.21% for the control group. The G minor allele frequency for PCOS and control groups was 60.53% and 54.79%, respectively. Contrary to our results, rs17817449 was not associated with PCOS risk in an American case-control cohort [23]. The frequency of G minor allele was 45.3% in the American population [23] compared to 60.53% in the PCOS group in the present study. Recently, in an Iraqi study evaluating the association of rs17817449 with obesity in PCOS women, the allele frequencies for T and G in PCOS women were, respectively, 48% and 52%, different from our findings [40].

In rs1421085, the allele frequency in the PCOS group of C (minor) and T (major) alleles was 51.9% and 48.1%, respectively, yet in the control group, it was 57.14% and 42.86%. In the American study, the minor allele frequency was 45.0% [23]. In a Chinese study [41], the frequencies of the rs1421085 genotypes in 212 PCOS women were 2.4% for C/C, 19.8% for C/T, and 77.8% for T/T compared to 16.7%, 70.9%, and 12.66%, respectively, in the PCOS group in our study. We found that the A minor allele frequency for rs8050136 in the PCOS group was 46.84%, similar to that in an American cohort [23]. Whereas, the genotype frequencies of rs8050136 variants were variable in the present study: 23.16% (A/A), 47.37% (A/C) and 29.47% (C/C), compared to 2.8%, 20.8%, and 76.4% respectively, in the Chinese population among PCOS patients [41].

We detected a significant association between PCOS susceptibility and the presence of rs17817449 and rs1421085 but not rs8050136. Several studies have examined the correlation and association between FTO variants and PCOS in other countries. For example, consistent with the present study, FTO variants rs17817449 and rs1421085 were found to be associated with PCOS susceptibility in the United Kingdom, China, Korea and recently, Sri Lanka [29, [31,42,43]. These findings indicate that FTO gene variants rs17817449 and rs1421085 could have a positive impact on the development of PCOS by increasing obesity. Similar to our results, a Brazilian study reported no association between the FTO rs8050136 variant and PCOS susceptibility [44]. However, in contrast, a Korean study found that the rs8050136 variant was associated with PCOS [31]. We believe that this discrepancy in findings could be attributed to ethnic variations and environmental factors.

The FTO variants rs17817449 and rs8050136 were correlated with loss of hair in PCOS women in our cohort. These results are supported by a Korean study that showed a significant association between these two variants and hyperandrogenemia in which loss of hair is one of the clinical manifestations [31,45]. In addition, a significant association was detected between the rs8050136 variant and elevated BMI (>30 kg/m2), which was also detected in the American study [23]. The risk genotype C/C in rs8050136 was associated with hair loss and high BMI in PCOS patients. However, the A allele was anticipated to be a global risk allele associated with high BMI and T2DM, based on previous studies [22,35,36]. The fact that high BMI and loss of hair are associated with the same risk allele (C) might confirm results from previous studies that suggest that obesity in PCOS women increases the risk of HA and its related clinical features [46,47].

## Conclusion:

The presence of FTO variants rs17817449 and rs1421085 are correlated with PCOS. Furthermore, the C/C genotype in rs8050136 is linked with hair loss and elevated BMI. Thus, the FTO variants are either directly or indirectly responsible for the progression of PCOS in addition to their previously known role in obesity. Therefore, the impact of FTO variants in a larger sample size in other regions of the Kingdom of Saudi Arabia is important.

## Funding:

This study was funded by the CIPM, King Abdulaziz University, Jeddah, Kingdom of Saudi Arabia.

## Authors'contributions:

S.B. and M.H.A. conceptualised the study. O.S.B. was the obstetrician/gynaecologist who diagnosed the patients. A.A.B., N.F. and A.D. performed experiments and analysed data. A.A.B., M.H.A. and S.B. wrote the manuscript. S.B. corrected the final version of the manuscript. All authors have read and approved the final manuscript.
